# If I told you that there is no need for yellow fever vaccine booster would you still come to the travel clinic?: a cross-sectional study

**DOI:** 10.1186/s40794-021-00132-8

**Published:** 2021-03-12

**Authors:** Iolanda Alves, Rosa Teodósio, Filomena Pereira

**Affiliations:** Global Health and Tropical Medicine, GHTM, Instituto de Higiene e Medicina Tropical, IHMT, Universidade Nova de Lisboa, UNL, Lisboa, Portugal

**Keywords:** Travelers, Travel health consultation, Prevention, Yellow fever vaccine, International health regulation amendment

## Abstract

**Background:**

Yellow Fever (YF) immunization required a single dose vaccine with boosters every 10 years. After International Health Regulation (IHR) amendment annex 7 (July 2016), it was accepted that a single dose confers lifelong immunity. Since pre-travel advice is as important as vaccination when traveling, it is essential to clarify why travelers come to a travel health consultation, with the possibility of IHR amendment having a negative impact on travelers’ health. This study aims to describe travelers’ reasons to come to a pre-travel consultation in Lisbon and if they would return if they wouldn’t need the YF vaccine booster.

**Methods:**

An observational cross-sectional study was conducted during 5 months in the waiting room of Instituto de Higiene e Medicina Tropical travel clinic in Lisbon, Portugal. Travelers were asked about sociodemographic characteristics, destination country, travel duration and reasons to travel in an anonymous self-administered questionnaire.

**Results:**

A total of 1043 travelers agreed to participate in the study. Although 61.0% (627/1028) did not come to the clinic to get the YF vaccine, from those who did, 36.7% (133/362) would not come and 12.9% (47/362) didn’t knew if they would come if the vaccine would not be necessary.

**Conclusion:**

The IHR amendment may have a negative impact on travel clinic attendance and on travelers´ health.

## Background

Yellow Fever (YF) is endemic in 34 countries in Africa and 13 in Central and South America [1]. Since the late 30’s prevention is possible, with the availability of the 17-D strain vaccine [2, 3]. YF vaccine is recommended for people aged 9 months or older who are travelling to or living in areas at risk of YF virus transmission. Furthermore, proof of YF vaccination is required by some countries to prevent travelers who visit endemic countries from introducing the virus into non-endemic countries [1, 4]. From the 63 countries endemic for YF, only 21 require proof of YF vaccination as a condition of entry [5].

Until recently, WHO recommendations for YF immunization were a single dose vaccine with booster doses every 10 years [4]. In April 2013, the WHO Strategic Advisory Group of Experts (SAGE) concluded that there is no need for booster doses, since a single dose of YF vaccine confers lifelong immunity in most individuals. Consequently, after the IHR amendment annex 7, on 11th July 2016, the International Certificate of Vaccination or Prophylaxis is considered valid for life. However, infants or immunocompromised patients might need to receive a second primary dose, a booster dose or serological monitoring [6, 7].

In addition to vaccination, pre-travel health advice is crucial to reduce the risk of infections while travelling [8]. Despite its importance, pre-travel advice is not mandatory and only a small number of travelers attend pre-travel clinics, mainly because they are not aware of travel health risks [9–11].

Hence, we think that it is important to clarify what motivates travelers to come to a pre-travel health consultation, especially when they only come to get the YF vaccine. In this case, travelers that wouldn’t need to get the YF vaccine lose a unique opportunity to be advised on the destination specific risks of infection and ways to prevent it. There would then be a possibility of the IHR amendment having a negative impact on travelers’ health.

Therefore, the objectives of this study were to assess: a) reasons for travellers to come to the Associação para o Desenvolvimento da Medicina Tropical (ADMT) /Instituto de Higiene e Medicina Tropical (IHMT) travel clinic in Lisbon; and b) their willingness to come to pre-travel health consultation if YF vaccination is not needed.

To the authors´ best knowledge, this is the first study that addresses both travelers’ motivations to come to a travel clinic and the impact that the IHR amendment could have on travelers not attending travel clinics.

## Methods

An observational cross-sectional study was conducted from May to September 2016 in ADMT/IHMT travel clinic in Lisbon, Portugal. The Angola YF outbreak occurred from January 21st to August 26th 2016, a period in which there was a great influx of travelers to our clinic whose destination was Angola. The travelers in the waiting room who agreed to participate and were at least 18 years old, completed a self-administered questionnaire. Individuals were asked about their sociodemographic characteristics, destination country, travel duration and reasons to travel and answered to the following questions: are you coming to the travel clinic to get the YF vaccine? Would you still come to this consultation if you wouldn’t have to get the YF vaccine? What other reasons do you have to come? Are you coming to this consultation to know what precautions to take while traveling? Are you coming to get other vaccines than the one against YF? Are you coming to learn how to prevent some diseases that may exist in the country of destination?

The data obtained was analyzed using the statistical program IBM SPSS Statistics 24.

## Results

A total of 1043 travelers participated in the study, with 57.2% (596/1042) being males and 42.8% (446/1042) females. The average age was 37.7 years (*n* = 1043, SD = 12.88 years, min = 18 years, máx = 80 years). The majority, 95.7% (726/759), was traveling for less than 6 months and from those who traveled for up to this length of stay, the average duration was 19.47 days (SD = 26.05 days, min = 1 day, max = 182 days, P_25_ = 7.0 days, P_50_ = 12.0 days, P_75_ = 19.0 days).

Leisure or adventure were the reasons to travel for 57.1% (590/1033) of the studied group, while work or business and visiting friends and relatives were for 46.6% (481/1033) and 10.5% (108/1033), respectively. The destination in 50.7% (505/997) was a country with risk of yellow fever (Table 1).

**Table 1:** Travelers’ sociodemographic and travel profile

	*n*.°/total n.°	%
Gender		
Male	596/1042	57.2
Female	446/1042	42.8
Age (y) (mean: 37.7y)		
18–29	344/1043	33.0
30–39	287/1043	27.5
40–49	207/1043	19.8
50–59	126/1043	12.1
≥ 60	79/1043	7.6
Planned journey length (d)		
< 6 months	726/759	95.7
≥ 6 months	33/759	4.3
Destination country		
With YF	505/997	50.7
Without YF	492/997	49.3
Reasons to travel*		
Work or business	481/1033	46.6
Adventure	154/1033	14.9
Leisure	436/1033	42.2
Visiting friends and relatives	108/1033	10.5
Other	50/1033	4.8

* Travelers could choose more than one reason to travel

**Fig. 1:**
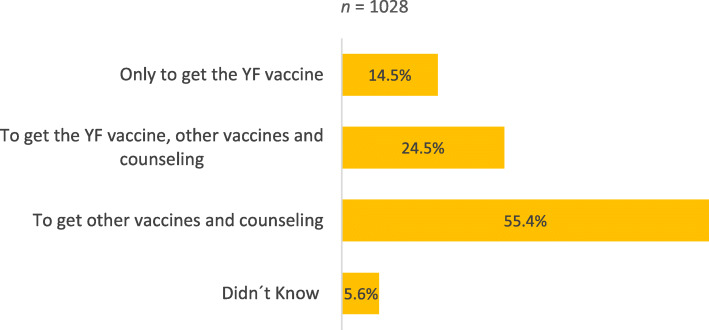
Travelers’ reasons to come to the clinic

**Fig. 2:**
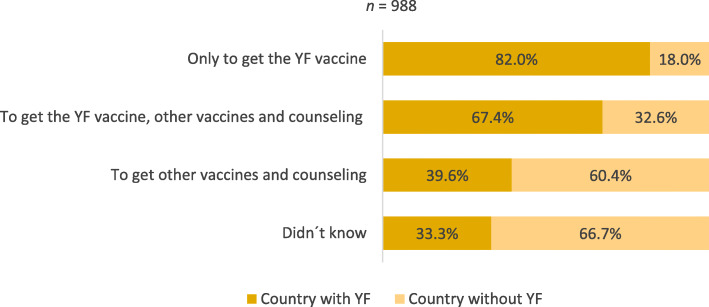
Travelers’ reasons to come to the clinic and country destination

**Fig. 3:**
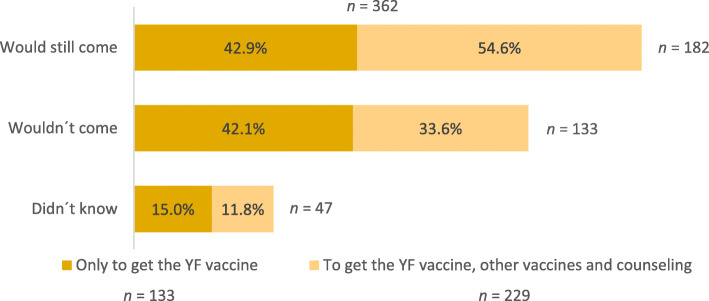
Travelers’ willingness to attend the clinic, if they wouldn’t need to take the YF vaccine

In relation to the reason for attending the clinic, 14.5% (149/1028) travelers came only to receive the YF vaccine and 24.5% (252/1028) to receive the YF vaccine, as well as other vaccines and counseling (Fig. 1). The majority of travelers who came to the travel health consultation motivated to: 1) receive the YF vaccine alone 82.0% (114/139); 2) receive concurrently the YF vaccine, other vaccines and counseling 67.4% (161/239), were travelling to countries where the vaccine was required (Fig. 2). When the travelers attending the travel clinic only to receive the YF vaccine were asked if they would still come to this consultation if the YF vaccine booster was no longer required, 42.9% (57/133) said they would still come and 42.1% (56/133) said they would not. From the group of travelers who came to the travel clinic to get the YF vaccine but also to seek counseling, 54.6% (125/229) would return and 33.6% (77/229) would not (Fig. 3).

## Discussion

To our knowledge, this is the first publication addressing the effect that YF lifelong immunity could have on travelers’ intentions to attend travel clinics.

In a pre-travel health consultation, a specialist evaluates the travel health risks of individuals, according to their medical history, destination, journey length, time of the year and intended activities. He/she also advises about risks, vaccination and prophylactic medication. Most diseases that develop while traveling can be prevented through these measures, although a high percentage of travelers are unaware of the travel health risks and as a result do not seek medical advice [10, 11]. It is also known that 64–76% of travelers have health problems during and 26–32% after traveling [9, 12, 13]. The medical community understands the importance of pre-travel consultation in preventing the diseases of the destination country that may become an individual and public health problem [14]. Studies on the cost effectiveness of travel consultation versus morbidity and mortality associated with transmission and spread of these diseases are difficult to perform. However, it is not difficult to understand that if for any reason attendance to travel clinics would decrease, the burden of such diseases would increase.

YF vaccine is reported as one of the most prescribed travel-associated vaccines [15]. It appears that some travelers attending our clinic mainly or solely needed the YF vaccine; therefore, we conducted this study to understand reasons for travellers to come and if they were still willing to come after knowing that yellow fever vaccination confers lifelong immunity.

Our travel clinic at the ADMT/IHMT in Lisbon is unique due to its history, liaison with travelers going to Portuguese speaking countries, some of them requiring the administration of YF vaccine and because it annually receives more travelers than all other travel consultations in the country together: for example, both in 2017 and 2018, there were above 11,000.

Consistent with other authors findings, the majority of participants in our study were traveling because of work and a very few were going to see friends or relatives [9, 11, 16, 17].

In our clinic, a high number of travelers (39.0%) stated that they came to the travel health consultation to get the YF vaccine, either as the only reason (14.5%) or also for other reasons (24.5%).

As expected, most travelers coming to the travel health consultation to do the YF vaccine were travelling to a country with YF. Still, travelers were unclear about their destination risks since 20 to 30% of the travelers who came to get the YF vaccine were traveling to a non-endemic country and the one-third that were traveling to a YF-endemic country, did not intend to receive the YF vaccine. Travelers that sought pre-travel advice along with YF vaccination were more willing to return to the travel clinic than the travelers seeking solely the YF vaccine. Therefore, the great majority of travelers interested in counseling would probably continue to attend the travel clinic. Nevertheless, half of the travelers would not return to the travel health consultation or didn’t know if they would return if YF vaccine booster were no longer needed.

Our results are consistent with those showing a decrease of 32.3% in the attendance of three travel clinics in France, after SAGE recommendations on YF vaccination [18, 19].

In this study, half of the travelers had a country with YF as their destination and the main reason for traveling was work and business. It is possible that some travelers were sent to the travel clinic by their employer, which may explain the lack of information regarding the motive for their travel clinic visit. Hence, the companies sending their workers abroad may have a critical role in motivating employees to attend pre-travel consultations regardless of the need for YF vaccination [20].

It should be noticed that our study was conducted in part during Angola’s YF outbreak and we think that if there was no outbreak, the number of travelers seeking the YF vaccine could be different. Furthermore, some individuals who came for the YF vaccine did so to get a vaccine booster (oral communication), which means that they had already been at a travel clinic and already know about some of the risks inherent to travel, although risks may vary from country to country and even in the same country.

## Conclusion

Although most travelers did not attend the travel health consultation at IHMT to seek for the YF vaccine, from those who did, half would probably not come if they wouldn’t need to get the vaccine.

The results obtained in this study suggest that while travelers have some notion of the country destination risks, the IHR amendment may have a negative impact on travelers´ health. It is our conviction that when travelers become aware that the International Certificate of Vaccination or Prophylaxis is valid for life, a large percentage will fail to come to the travel clinic and receive advice that is crucial to reduce the risk of infections while traveling.

These individuals will probably travel without pre-travel consultation, losing the opportunity of being advised and getting other vaccines that could be necessary in accordance with their destination. This will put them at risk of being infected with diseases that could be transmitted to other individuals, to pregnant women and their newborns, such as Zika virus infection and others or even introducing a viral agent in a country where its specific vector exists.

## Data Availability

Not applicable.

## Abbreviations

ADMT: Associação para o Desenvolvimento da Medicina Tropical
IHMT: Instituto de Higiene e Medicina Tropical
IHR: International Health Regulations
SAGE: Strategic Advisory Group of Experts
YF: Yellow Fever
